# Supplementary material for: Resolving the full spectrum of human genome variation using Linked-Reads
Source: Genome Res. 2019 Apr;29(4):635–45. doi: 10.1101/gr.234443.118 (PMC6442396; doi:10.1101/gr.234443.118)
Supplement: Supplemental Material [file supp_gr.234443.118_Supplemental_longranger-2.2.zip › longranger-2.2.tar.bz2!/lr2.2-public/lib/stan/cmdstan/stan/doc/api/html/fwd_2functions_2pow_8hpp_source.html]

Stan: src/stan/agrad/fwd/functions/pow.hpp Source File


|  |  |
| --- | --- |
|  | Stan  2.5.0  probability, sampling & optimization |


- Main Page
- Related Pages
- Namespaces
- Classes
- Files

- File List
- File Members

All Classes Namespaces Files Functions Variables Typedefs Enumerations Enumerator Friends Macros Pages

- src
- stan
- agrad
- fwd
- functions

pow.hpp

Go to the documentation of this file.

1 #ifndef STAN\_\_AGRAD\_\_FWD\_\_FUNCTIONS\_\_POW\_HPP

2 #define STAN\_\_AGRAD\_\_FWD\_\_FUNCTIONS\_\_POW\_HPP

3

4 #include <stan/agrad/fwd/fvar.hpp>

5 #include <stan/meta/traits.hpp>

6 #include <stan/agrad/fwd/functions/inv.hpp>

7 #include <stan/agrad/fwd/functions/inv\_sqrt.hpp>

8 #include <stan/agrad/fwd/functions/inv\_square.hpp>

9

10 namespace stan {

11

12  namespace agrad {

13

14  template <typename T>

15  inline

16  fvar<T>

17  pow(const fvar<T>& x1, const fvar<T>& x2) {

18  using std::pow;

19  using std::log;

20  T pow\_x1\_x2(pow(x1.val\_,x2.val\_));

21  return fvar<T>(pow\_x1\_x2,

22  (x2.d\_ \* log(x1.val\_)

23  + x2.val\_ \* x1.d\_ / x1.val\_) \* pow\_x1\_x2);

24  }

25

26  template <typename T>

27  inline

28  fvar<T>

29  pow(const double x1, const fvar<T>& x2) {

30  using std::pow;

31  using std::log;

32  T u = pow(x1,x2.val\_);

33  return fvar<T>(u, x2.d\_ \* log(x1) \* u);

34  }

35

36  template <typename T>

37  inline

38  fvar<T>

39  pow(const fvar<T>& x1, const double x2) {

40  using std::pow;

41  using stan::agrad::inv;

42  using stan::agrad::inv\_sqrt;

43  using stan::agrad::inv\_square;

44  using std::sqrt;

45  using stan::math::square;

46

47  if (x2 == -2)

48  return inv\_square(x1);

49  if (x2 == -1)

50  return inv(x1);

51  if (x2 == -0.5)

52  return inv\_sqrt(x1);

53  if (x2 == 0.5)

54  return sqrt(x1);

55  if (x2 == 1.0)

56  return x1;

57  if (x2 == 2.0)

58  return square(x1);

59

60  return fvar<T>(pow(x1.val\_, x2),

61  x1.d\_ \* x2 \* pow(x1.val\_, x2 - 1));

62  }

63  }

64 }

65 #endif

stan::math::square

T square(const T x)

Return the square of the specified argument.

**Definition:** square.hpp:22

inv\_sqrt.hpp

inv.hpp

stan::agrad::pow

fvar< T > pow(const fvar< T > &x1, const fvar< T > &x2)

**Definition:** pow.hpp:17

stan::agrad::fvar

**Definition:** fvar.hpp:13

stan::agrad::square

fvar< T > square(const fvar< T > &x)

**Definition:** square.hpp:15

traits.hpp

stan::agrad::inv

fvar< T > inv(const fvar< T > &x)

**Definition:** inv.hpp:15

inv\_square.hpp

stan::agrad::inv\_square

fvar< T > inv\_square(const fvar< T > &x)

**Definition:** inv\_square.hpp:15

stan::agrad::sqrt

fvar< T > sqrt(const fvar< T > &x)

**Definition:** sqrt.hpp:15

fvar.hpp

stan::agrad::pow

var pow(const double base, const var &exponent)

Return the base scalar raised to the power of the exponent variable (cmath).

**Definition:** pow.hpp:144

stan::agrad::fvar::d\_

T d\_

**Definition:** fvar.hpp:16

stan::agrad::log

fvar< T > log(const fvar< T > &x)

**Definition:** log.hpp:15

stan::agrad::inv\_sqrt

fvar< T > inv\_sqrt(const fvar< T > &x)

**Definition:** inv\_sqrt.hpp:15

stan::agrad::fvar::val\_

T val\_

**Definition:** fvar.hpp:15


---

[ Stan Home Page ]

*© 2011–2014,
Stan Development Team.*
